# Perception and barriers to electronic medical record use among physicians and nurses in General Hospitals in Lagos State

**DOI:** 10.4314/ahs.v25i3.20

**Published:** 2025-09

**Authors:** Olasimbo A Babalola, Tolulope F Olufunlayo, Olamide O Akinlawon, Temiloluwa O Odukoya, Oluwabusayo O Fagbo, Damilola Akinlawon

**Affiliations:** 1 Department of Community Health and Primary Care, Lagos University Teaching Hospital; 2 Department of Community Health and Primary Care, College of Medicine, University of Lagos; 3 Federal Neuropsychiatric Hospital, Yaba, Lagos

**Keywords:** Electronic Medical Records, Perception, Barriers, Physicians, Nurses

## Abstract

**Background:**

The adoption of Electronic Medical Records (EMR) in Nigeria has been slow despite the benefits. Health worker perception is pertinent in promoting its adoption.

**Objectives:**

To assess the perception and barriers to EMR use among physicians and nurses in general hospitals in Lagos, Nigeria.

**Methods:**

This was a descriptive cross-sectional study utilizing multistage sampling to select 293 respondents. Data was collected using a self-administered questionnaire, analysed and presented as frequency tables. Chi-squared test determined association with EMR perception with level of significance at p ≤ 0.05.

**Results:**

The mean age of the respondents was 36.6 ± 10.2 years. Majority of the respondents (69.3%) were female. Most respondents (98.6%) had positive perception of EMR. Major barriers to the use of EMR highlighted by the respondents included insufficient computers (90.8%), inconsistent power supply (87.0%), hardware or software failure (85.7%), and poor internet (84.7%). There was significant association between respondents age group and EMR perception (p = 0.018).

**Conclusion:**

There was a predominantly positive perception of EMR among healthcare workers in Lagos, despite significant barriers such as inadequate infrastructure and inconsistent power supply. Strategic investments in technology, reliable electricity, and internet connectivity are needed for enhancing EMR adoption and optimizing healthcare delivery.

## Introduction

The importance of documentation and record-keeping in medicine has been recognized since its earliest practices, evolving over time from manual paper and pen to modern digital solutions^1^. The adoption of electronic medical records (EMR), a key component of Health Information Technology (HealthIT), represents a significant shift in healthcare^2^. Primarily utilized by physicians and nurses in the healthcare setting, EMR adoption has become a global standard for improving healthcare services, reducing errors, enhancing safety, and ensuring effective care delivery^3^-^5^.

Globally, the adoption of EMR systems is spearheaded by developed nations, where centralized databases enable clinicians to access patient data efficiently. According to a WHO survey, 114 countries are working toward national EMR systems to strengthen HealthIT and provide cost-effective care^6^. Despite the benefits of EMR, including reduced waiting times, improved documentation legibility, and space efficiency in hospitals, adoption rates remain low in Nigeria, with only 18–23% of hospitals using EMRs and a mere 15% utilizing computerized physician order entry systems^7^.

This adoption rate places Nigeria behind some African counterparts. In Kenya, EMR adoption among healthcare facilities has been reported to range between 24% and 46%, depending on the level of care, with teaching and referral hospitals having higher adoption rates^3^. Similarly, Ethiopia has seen significant progress, with EMR adoption rates varying between 19% and 35% in different regions^8^. However, despite these relatively higher adoption rates in some parts of Africa, EMR utilization remains a challenge due to infrastructure constraints, lack of training, and resistance to change. This contrasts sharply with the United States, where EMR adoption exceeds 96% in non-federal acute care hospitals^9^.

Healthcare providers' perceptions significantly influence the adoption and utilization of EMR systems. Positive perceptions, such as ease of use and usefulness, promote adoption, while negative perceptions, including low computer literacy, fear of technology, and dissatisfaction, hinder utilization.^5^ Internationally, studies in the United States, Europe, and Saudi Arabia, underscore the significance of user perceptions in shaping EMR adoption. While perception was generally positive in these regions, there were still health care providers that showed resistance to EMR utilization. Common barriers highlighted in these populations included confidentiality and privacy, system usability issues, and workflow disruptions, with training emerging as a critical factor for improving satisfaction and usage^9^-^14^.

In sub-Saharan Africa, the adoption of EMR has been met with mixed perceptions^3^,^8^,^15^,^16^. Factors such as inadequate infrastructure, lack of training, and resistance to change significantly affected EMR adoption. In Kenya, for instance, a study highlighted that healthcare providers recognized the potential benefits of EMR but were hindered by technical and organizational barriers^3^. Similarly, in Ethiopia, common barriers included limited technical support and inadequate training programs^8^.

Studies conducted across Nigeria also reveal varying perceptions among healthcare providers though majority of health workers generally had positive perception towards EMR^4^,^17^-^18^. A study in Plateau state in Nigeria showed a majority of nurses (76.8%) with positive perception towards EMR, however majority of them (75.4%) were not utilizing the technology^4^. This could be attributed to the barrier of lack of training and paucity of sufficient equipment as seen in another study in Osun state where as many as 79.6% had positive perception and were willing to utilize EMR but a vast majority (80.1%) had never used the available health records technologin the facility^17^. Another study in Kaduna showed most of the health workers (96.9%) had positive perception of EMR^18^.

In Lagos State, limited research exists on the perceptions and barriers to EMR adoption among healthcare professionals, particularly in general hospitals. A study performed in the federal teaching hospital located in the state, showed a 100% positive attitude and 96.54% willingness to use EMR among the doctors^19^. The significance of this study lies in its focus on the perceptions and barriers towards the utilization of this transformative health tool by physicians and nurses in Lagos, who are the primary users of EMR systems.

This study aimed to assess the perception and barriers to EMR use among physicians and nurses in general hospitals in Lagos, Nigeria. This goes beyond assessing willingness to adopt EMR by examining the real-world challenges faced by physicians and nurses using these systems in Lagos general hospitals. Unlike previous research based on hypothetical perspectives, it captures firsthand experiences, highlighting key barriers such as infrastructure deficiencies, technical issues, and workflow disruptions. These insights are essential for developing targeted interventions that address both adoption and implementation challenges. By identifying the level of positive perception and barriers, this study has implications not only for Lagos State but also for Nigeria's broader healthcare ecosystem, providing insights that could inform strategic policy decisions and resource allocation for the digital transformation of healthcare delivery.

## Methods

### Study location

Lagos State, one of Nigeria's 36 states, is the second most densely populated, with an estimated population of over 22 million. It is divided into five administrative divisions—Ikeja, Badagry, Ikorodu, Lagos Island (Eko), and Epe — which are further split into 20 Local Government Areas (LGAs). The state covers 3,577 square kilometers, with a mix of urban and peri-urban settlements, making it a key economic and healthcare hub in Nigeria^20^.

Lagos has a relatively well-developed healthcare system, comprising 2,333 public and private health facilities, including primary, secondary, and tertiary institutions. Specifically, there are 412 public primary facilities, 1,162 private primary facilities, 44 public secondary facilities, 712 private secondary facilities, two public tertiary facilities, and one private tertiary facility^21^.

The state operates 28 general hospitals with 16 general hospitals currently using electronic medical records (EMR) systems. General hospitals are public hospitals offering secondary care in Lagos and have a uniform array of services and capacity, offering outpatient and inpatient care, emergency services, and specialized care, with only minor differences in personnel, infrastructure, and logistics which are mostly due to the hospital locations^20^.

### Study Design and Population

This is a descriptive cross-sectional study to assess the perception and barriers to electronic medical record use among physicians and nurses in selected general hospitals in Lagos State.

### Inclusion criteria

Physicians and nurses who worked in general hospitals that use electronic medical record systems.

Physicians and nurses who had used electronic medical record systems for more than 3 months.

### Exclusion criteria

Physicians and Nurses who worked in administrative positions and not clinical positions in the selected general hospitals.

### Sample Size Determination

The sample size was determined using Cochran's Formula; n = Z2pq/d2, where; Z is Standard normal deviate at 95% confidence interval which is 1.96, p is the proportion of respondents with positive perception of EMR in a previous study which was 76.8%.4, q = complement probability which is 1-p, d is the measure of precision or error margin put at 5% (0.05) for the study, and n is the minimum sample size to be calculated.

Inputting the values we see that n = 273.76 = 274.

As of January 2024, according to the Lagos State Ministry of Health, Digital Health Unit, the combined population of physicians and nurses in general hospitals in Lagos State stands at 4,521. Consequently, there is a need for a finite correction factor; nf = n/[1 + (n/N)], where n is the minimum sample size, 274, N is the study population size, 4,521, nf is the corrected sample size. Inputting the values we see that n = 258.5 = 259.

10% of the minimum sample size was added for nonresponse:

Therefore the minimum sample size = 284.9 = 285.

### Sampling Technique

Multistage sampling method was used to select the respondents involved in the study.

Stage 1 (selection of hospitals): There were 16 general hospitals under the five administrative divisions of Lagos, which utilize EMR technology. Five (5) general hospitals were selected out of the sixteen (16) hospitals by simple random sampling (balloting).

Stage 2 (determination of number of respondents to be selected from each hospital): Allocation of respondents in each hospital was acquired using the formula;

(Nx/Nt) * minimum sample size; where, Nx is the total number of physicians and nurses in a hospital, Nt is the total number of physicians and nurses in the five general hospitals selected.

Stage 3 (selection of respondents): The respondents were selected for the study using a systematic sampling method from the sampling frame which was the list of physicians and nurses that was gotten from the administrative department of the hospitals.

The sampling interval (k) is determined using the formula:


$$k=\frac{Total\;number\;of\;physicians\;and\;nurses\;in\;the\;hospital\;(N)}{Sample\;size\;required\;from\;the\;hospital\;(n)}$$

This interval (k) indicates the gap between the selected individuals from the sampling frame. The first respondent was selected by simple random sampling (balloting).

### Method Of Data Collection

Data collection was carried out using a pretested, structured, self-administered questionnaire adapted from previous studies^4^,^13^,^15^,^18^,^19^ which comprised of Likert scale questions. The questionnaire was divided into three sections: Section A contains the Socio-demographic characteristics of the respondents, which included age as at the last birthday, gender which will be male or female, this is according to Nigeria's cultural context, and profession (physician or nurse). Section B contained the perception of electronic medical records among respondents. Section C contained the perceived barriers in electronic medical records use among respondents. The questionnaire was pretested at Shomolu General Hospital, then the data collected was retrieved and the questionnaire was reviewed and checked for clarity to make necessary adjustments or modifications.

### Data Analysis

Data was entered into Microsoft Excel 365 and then imported for analysis into IBM SPSS version 20. The data was presented as frequency tables. Chi-square test was used to determine significant association at a significance level of p ≤ 0.05.

A Likert 5-point scale was used to assess the perception of EMR use, and scores given to all 22 questions were based on their level of agreement or disagreement. Five marks was allocated for every Strongly Agree, four marks for Agree, three marks for Neutral, two marks for Disagree, and one mark for Strongly Disagree. Negative statements were reversely scored during the analysis. Each respondent could potentially score a minimum of 22 marks and a maximum of 110 marks. A mid-point of 66 (between 22 and 110) was used to determine which respondents have a positive perception towards EMR use, and those that have a negative perception towards EMR use. Respondents who scored 66 and above were said to have a positive perception while those who scored less than 66 were said to have a negative perception.

### Ethical Consideration

Ethical approval for the study was obtained from the Health Research Ethics Committee (HREC) of the Lagos University Teaching Hospital, with the HREC assigned number: ADM/DSCST/HREC/APP/6621. Permission was sought from the Managing Director (MD)/Chief Executive Officer (CEO) of each general hospital after approval from the Lagos State Health Service Commission (HSC) was obtained. Informed consent was obtained from the participants before their enrolment, and strict confidentiality of all participant's information was ensured by not putting any form of identifier on the questionnaire and using numbers to identify the questionnaires. Participation in the study was entirely voluntary, participants chose to take part without any coercion and could withdraw at any time without any consequences.

## Results

This study involved physicians and nurses in general hospitals in Lagos State. Out of the 300 questionnaires administered, 293 were returned and filled, yielding a 97.7% response rate. The mean age of the respondents was 36.6 ± 10.2 years, ranging from 22 to 67 years. The highest proportion of respondents (41.6%) were within the age group of 30 to 39 years. More than two-thirds (69.3%) were female, and just over half (53.9%) were nurses. The mean number of years of experience among respondents was 10.4 ± 8.8 years, with a range of 1 to 40 years (Table 1).

**Table 1:** Socio-demographic characteristics of respondents

Variable	Frequency (n=293)	Percentage (%)
**Age group (years)**		
20-29	78	26.6
30-39	122	41.6
40-49	50	17.1
≥50	43	14.7
**Range**	22-67	
**Mean± SD**	36.6± 10.2	
**Sex**		
Male	90	30.7
Female	203	69.3
**Profession**		
Physicians	135	46.1
Nurses	158	53.9
**Years of Experience (years)**		
≤5	125	42.7
6-10	57	19.5
11-15	43	14.7
16-20	31	10.6
≥20	37	12.6
**Range**	1-40	
**Mean± SD**	10.4± 8.8	

Regarding perceptions of electronic medical records (EMR), the majority strongly agreed that EMRs improved access to patient health information compared to paper records (53.2%), eliminated issues of illegible handwriting by healthcare providers (59.7%), and facilitated easier collation and analysis of hospital statistics (60.8%). Almost half (42.0%) strongly agreed that EMRs should replace paper-based records. Additionally, the highest proportion of respondents agreed that EMRs would lead to faster patient care (44.4%), allow for attending to more patients daily (42.7%), reduce patient waiting time (38.9%), improve healthcare performance (45.4%), enhance communication between healthcare personnel (45.1%), reduce errors in healthcare (44.0%), and improve patient care and safety (50.5%). A majority also agreed that EMRs should be used in routine patient care (47.1%) and that the system was easy to learn (52.5%). Additionally, 47.8% found information from EMRs to be accurate and easy to understand, and 46.4% believed EMRs were better than paper-based records. However, 31.9% felt EMRs should be used alongside paper records, and 37.9% perceived poor cultural acceptability, particularly among the older generation (Table 2).

**Table 2:** Perception of Electronic Medical Records use among respondents

Variable (n = 293)	Strongly Disagree	Disagree	Neutral	Agree	Strongly Agree
EMR would make patient care faster than paper	10(3.4)	19(6.5)	22(7.5)	130(44.4)	112(38.2)
EMR should be used in the routine care of patients	5(1.7)	4(1.4)	17(5.8)	138(47.1)	129(44.0)
The EMR system is easy to learn	4(1.4)	15(5.1)	27(9.2)	154(52.5)	93(31.7)
EMR should only be used for specialized patient care	88(30.0)	141(48.1)	20(6.8)	21(7.2)	22(7.5)
EMR helps to attend to more patients daily	12(4.1)	36(12.3)	47(16.0)	125(42.7)	73(24.9)
EMR reduces patient waiting time.	6(2.0)	50(17.1)	44(15.0)	114(38.9)	79(27.0)
EMR will help patients easily access and retrieve their health information.	6(2.0)	3(1.0)	5(1.7)	123(42.0)	156(53.2)
Patient's privacy and confidentiality will be insecure with EMR	63(21.5)	104(35.5)	44(15.0)	44(15.0)	38(13.0)
EMR reduce patients' cost of health services.	43(14.7)	118(40.3)	70(23.9)	40(13.7)	22(7.5)
EMR allow seamless communication between healthcare personnel.	12(4.1)	20(6.8)	32(10.9)	132(45.1)	96(32.8)
Documentation, consultation, prescription, dispensing of drugs, and appointment system with the use of EMR will improve performance compared to paper-based records	4(1.4)	11(3.8)	21(7.2)	133(45.4)	124(42.3)
EMR system usage can reduce medication & other errors	7(2.4)	33(11.3)	52(17.7)	129(44.0)	72(24.6)
EMR take care of the illegible handwriting of healthcare providers	5(1.7)	2(0.7)	9(3.1)	102(34.8)	175(59.7)
The EMR system is useful	3(1.0)	1(0.3)	4(1.4)	116(39.6)	169(57.7)
EMR make hospital statistics collation & analysis easier.	3(1.0)	2(0.7)	10(3.4)	100(34.1)	178(60.8)
Information from the EMR system is accurate and easy to understand	2(0.7)	4(1.4)	32(10.9)	140(47.8)	115(39.2)
EMR are better than paper-based records	2(2.7)	2(0.7)	37(12.6)	136(46.4)	116(39.6)
EMR should replace paper-based records	9(3.1)	11(3.8)	38(13.0)	112(38.2)	123(42.0)
EMR should be used together with paper-based records	34(11.6)	50(17.1)	73(24.9)	93(31.7)	43(14.7)
EMR would lead to better patient care and safety	5(1.7)	10(3.4)	51(17.4)	148(50.5)	79(27.0)
EMR require too much time to train hospital staff on their use	29(9.9)	135(46.1)	45(15.4)	66(22.5)	18(6.1)
EMR have poor cultural acceptability, particularly among the older generation	30(10.2)	66(22.5)	44(15.0)	111(37.9)	42(14.3)

In contrast, most respondents disagreed that EMRs should only be used for specialized patient care (48.1%) or that EMRs compromised patient privacy and confidentiality (35.5%). Similarly, 40.3% disagreed that EMRs reduce healthcare costs, and 46.1% disagreed that EMRs require excessive staff training time (Table 2).

Several barriers to EMR use were identified, including an insufficient number of computers leading to slow documentation (90.8%), inconsistent power supply (87.0%), risk of data loss due to hardware or software failure (85.7%), lack of reliable and fast internet (84.7%), and lack of basic computer skills among users (77.1%). Additional challenges included inadequate ICT support (75.4%), lack of proper EMR training (75.1%), and frequent need for ICT assistance (65.5%). Less frequently cited barriers were time-consuming documentation (30.0%), an unfriendly user interface (16.4%), and general difficulty in using EMR technology (5.8%) (Table 3).

**Table 3:** Barriers to EMR use among respondents

Variable (n = 293)	Agree	Neutral	Disagree
Lack of proper technical training on the EMR software makes it difficult to use	220(75.1)	32(10.9)	41(14.0)
The constant need for ICT assistance when using EMR is a challenge	192(65.5)	49(16.7)	52(17.7)
Lack of technical support from the ICT department makes EMR use challenging	221(75.4)	36(12.3)	36(12.3)
EMR is generally a difficult-to-use technology	17(5.8)	29(9.9)	245(83.6)
Lack of basic computer skills can make EMR use difficult	226(77.1)	33(11.3)	33(11.3)
The inability to retrieve patient data when there is a hardware or software failure is a barrier to EMR use	251(85.7)	26(8.9)	16(5.5)
The EMR user interface is not user-friendly	48(16.4)	88(30.0)	157(53.6)
The inconsistent power supply leads to an unpleasant experience when using EMR	255(87.0)	15(5.1)	22(7.5)
Lack of reliable and fast internet makes EMR use frustrating	248(84.7)	13(4.4)	32(10.9)
Insufficient computers, with more users than available devices, in facilities result in slow documentation processes	266(90.8)	17(5.8)	8(2.7)
Documentation for patients with EMR is time-consuming	88(30.0)	56(19.1)	149(50.9)

Overall, almost all respondents (98.6%) had a positive perception of EMR use, while only four (1.4%) had a negative perception (Table 4). The four respondents with a negative perception were all within the age range of 20 to 29 years, female, nurses, and had less than five years of experience. There was a statistically significant association between age group and overall perception of EMR use (p = 0.018), while other socio-demographic factors such as sex, profession, and years of experience were not significantly associated with perception (Table 5).

**Table 4:** Overall Perception of EMR use among respondents

Perception	Frequency (n = 293)	Percentage (%)
Positive	289	98.6
Negative	4	1.4

**Table 5:** Association between the overall perception of EMR and socio-demographic of respondents

Variable	Positive Perception (n = 289)	Negative Perception (n = 4)	Chi-square	p-value/Fischer's exact
**Age group (years)**				
20-29	74 (94.9)	4 (5.1)	7.0	0.018* **
30-39	122 (100.0)	0 (0.0)		
40-49	50 (100.0)	0 (0.0)		
≥50	43 (100.0)	0 (0.0)		
**Sex**				
Male	90 (100.0)	0 (0.0)	1.8	0.316**
Female	199 (98.0)	4 (2.0)		
**Profession**				
Physicians	135 (100.0)	0 (0.0)	3.5	0.125**
Nurses	154 (97.5)	4 (2.5)		
**Years of Experience (years)**				
≤5	121 (96.8)	4 (3.2)	2.8	0.505**
6-10	57 (100.0)	0 (0.0)		
11-15	43 (100.0)	0 (0.0)		
16-20	31 (100.0)	0 (0.0)		
≥20	37 (100.0)	0 (0.0)		

* Statistically significant

** Fisher's Exact P-value

## Discussion

This descriptive cross-sectional study investigated the perceptions and barriers associated with the use of electronic medical records (EMR) among physicians and nurses in general hospitals in Lagos State, Nigeria. A high positive perception of EMR among physicians and nurses is an integral factor in successful healthcare digitization, as positive attitudes enhance user acceptance and engagement, driving the effective adoption of EMR systems^2^,^6^. It is therefore necessary that this form of assessment be made on the installation of healthcare digitization, as it would expose the propensity for uptake and sustainability or resistance in the health system it was set to be instituted. The findings in this study reveal a predominantly positive perception of EMR use (98.6%) among respondents, with some critical barriers impeding its adoption and utilization. This finding aligns with similar studies in Nigeria and other countries, which also revealed high positive perception among their health workers^9^,^16^-^19^.

Most respondents (82.6%) acknowledged that EMR expedites patient care compared to traditional paper records, emphasizing its role in improving healthcare efficiency. Additionally, 91.1% supported the routine use of EMR in hospitals, with majority (78.1%) rejecting the notion that EMR should be limited to specialized care. This broad endorsement underscores the perceived applicability of EMR across various clinical contexts^8^. Ease of use was another critical factor, with 84.2% of respondents agreeing that EMR systems are easy to learn. This finding contrasts with a study on nurses in Saudi Arabia that highlighted EMR as challenging to use despite being in a developed setting, underscoring the variability in user experience based on system design and implementation^22^. Notably, 67.6% believed EMR enhances the capacity to manage more patients daily, aligning with findings from tertiary healthcare facilities in southwestern Nigeria^23^.

Despite the positive perceptions, several barriers were identified. A lack of proper technical training (75.1%) and insufficient ICT support (75.4%) emerged as significant challenges. These findings mirror studies from both developed and developing health care settings; Europe and Ethiopia, emphasizing the need for capacity building and sustained technical assistance^12^,^14^. Hardware and software failures were also notable barriers across board, with 85.7% in this study reporting difficulties in retrieving patient data during system downtimes, concurring with studies from Hong Kong, Hawai'i and Egypt, emphasizing the need for robust backup systems to ensure continuity of care during technical disruptions^14^.

Concerns on security and privacy was the most frequently mentioned barrier to EMR adoption in many developed countries^14^. However, it was not a major issue highlighted by respondents in this study as more respondents disagreed (35.5%) and strongly disagreed (21.5%) that EMR utilization may breach privacy and confidentiality. Barriers that were more peculiar to the clime of health care systems in developing countries were also highlighted by respondents. They included infrastructural challenges such as inconsistent power supply (87.0%), insufficient number of computers to match available health workers (90.8%), and unreliable internet connectivity (84.7%), reflecting broader systemic weaknesses in Nigerian healthcare infrastructure. Furthermore, 77.1% of respondents cited insufficient basic computer skills as a challenge, suggesting a considerable level of computer illiteracy even among tertiary educated healthcare workers, and reinforcing the importance of general digital literacy alongside specialized EMR training^7^.

This study identified age as a significant factor influencing EMR perception (p = 0.018). All the respondents with negative perception were in the youngest age group, female, nurses and had their years of experience in the lowest range of five years and below. This finding contrasts with expectations that younger healthcare professionals, being more tech-savvy, would exhibit greater acceptance. Possible explanations include that the younger professionals may be more critical of EMR systems due to their higher expectations for seamless, user-friendly technology. They might also encounter more frustrations with system limitations or usability issues compared to their older counterparts, who may have had more time to adapt to and accept the system's imperfections. Additionally, this group is often at the forefront of direct patient care, where inefficiencies in documentation could directly impact their workflow and job satisfaction.

## Conclusion

This study revealed a significant majority of physicians and nurses working in general hospitals, held a positive perception of EMR. Barriers to EMR usage were also identified, including insufficient technical training and support, unreliable and slow internet connectivity, inconsistent power supply, and an inadequate number of computers. All the respondents with negative perception were at the youngest age group, which was significantly associated with EMR perception.

Recommendations would involve the intentional approach by stakeholders towards the adequate supply of training, IT support, power, internet connectivity and essential hardware and software for seamless provision of healthcare services. Also, tailored interventions to address the concerns of younger professionals and ensure system reliability will be pertinent in fostering widespread acceptance and improving healthcare delivery. Future research should explore long-term EMR impacts in longitudinal studies, intervention strategies, and/or regional adoption variations

### Strengths and limitations

This study provides valuable insights into the perceptions and barriers to EMR adoption among physicians and nurses in Lagos general hospitals, contributing to the limited literature on digital health in Nigeria. Its strength lies in capturing real-world challenges, offering practical recommendations for improving EMR implementation. However, the study is limited by its cross-sectional design, which prevents causal inferences. Additionally, self-reported data may introduce response bias, and findings may not be generalizable to private hospitals or other regions. Future studies with longitudinal designs and broader geographic coverage would strengthen understanding of EMR adoption trends and challenges across Nigeria.
